# A New Diketopiperazine, Cyclo-(4-*S*-hydroxy-*R*-proline-*R*-isoleucine), from an Australian Specimen of the Sponge *Stelletta* sp. †

**DOI:** 10.3390/md9112469

**Published:** 2011-11-22

**Authors:** Simon P. B. Ovenden, Jonathan L. Nielson, Catherine H. Liptrot, Richard H. Willis, Dianne M. Tapiolas, Anthony D. Wright, Cherie A. Motti

**Affiliations:** 1 Australian Institute of Marine Science, PMB no. 3, Townsville MC, Townsville 4810, Australia; E-Mails: simon.ovenden@dsto.defence.gov.au (S.P.B.O.); jonathon.nielson@acdlabs.com (J.L.N.); catherine.liptrot@jcu.edu.au (C.H.L.); r.willis@aims.gov.au (R.H.W.); d.tapiolas@aims.gov.au (D.M.T.); adwright@hawaii.edu (A.D.W.); 2 Defence Science & Technology Organisation, 506 Lorimer St. Fishermans Bend, Victoria 3207, Australia; 3 ACD Labs UK, Building A, Trinity Court, Wokingham Road, Bracknell, Berkshire RG42 1PL, UK; 4 Advanced Analytical Centre, James Cook University, Townsville, QLD 4811, Australia; 5 College of Pharmacy, University of Hawaii, 34 Rainbow Drive, Hilo, HI 96720, USA

**Keywords:** *Stelletta*, diketopiperazine (DKP), cyclo-(4-*S*-hydroxy-*R*-proline-*R*-isoleucine), bengamide, bengazole, anti-cancer activity

## Abstract

While investigating the cytotoxic activity of the methanol extract of an Australian marine sponge *Stelletta* sp. (Demospongiae), a new diketopiperazine, cyclo-(4-*S*-hydroxy-*R*-proline-*R*-isoleucine) (**1**), was isolated together with the known bengamides; A (**2**), F (**3**), N (**4**), Y (**5**), and bengazoles; Z (**6**), C_4_ (**7**) and C_6_ (**8**). The isolation and structure elucidation of the diketopiperazine (**1**), together with the activity of **1**–**8** against a panel of human and mammalian cell lines are discussed.

## 1. Introduction

Since the first reported isolation of a diketopiperazine (DKP) from the sponge *Dysidea herbacea* [1], there have been several reports describing the isolation of this class of compound from other marine sponges [2–4]. DKPs are also reported from marine microbial sources [5–8], including the proteobacteria *Alcaligenes faecalis*, isolated from the sponge *Stelletta tenuis* [9]. The metabolites reported in these investigations are mostly the products of 4-hydroxy-proline [2,5,6,8] or proline [7] reacting with phenylalanine [2,5], arginine [4], leucine [5–7], isoleucine [7], norvaline [3] or alanine [8].

Sponges from the genus *Stelletta* are known to produce a number of other bioactive classes of compounds, including but not limited to steroids [10], alkaloids [11,12], isomalabaricane triterpenes [13], acetylenic acids [14] and lysophosphatidylcholines [15]. Initial interest in the methanol (MeOH) extract of the sponge *Stelletta* sp. was motivated by to its potent activity in the NCI 60 cell line screen and a unique COMPARE analysis profile (average GI_50_ 0.5 μg/mL) [16]. This profile was different to that of the standard chemotherapeutic agents paclitaxel, cisplatin, gemcitadine, bryostatin 1, didemnin B, tamoxifen and vinblastine (data provided by NCI). Subsequent bioassay-guided investigations of this extract led to the isolation of a new DKP cyclo-(4-*S*-hydroxy-*R*-proline- *R*-isoleucine) (**1**), the previously reported bengamides; A (**2**) [17], F (**3**) [18], N (**4**) [19], Y (**5**) [20], and the previously reported bengazoles; Z (**6**) [20], C_4_ (**7**) [21] and C_6_ (**8**) [21]. Described in this publication is the isolation and structure elucidation of **1**, together with the activity of compounds **1**–**8**.

## 2. Results and Discussion

**Scheme 1:**
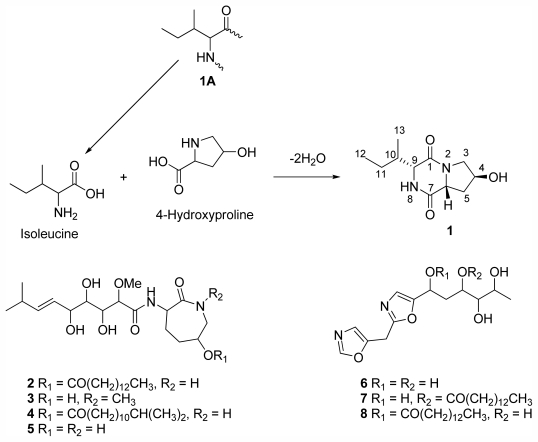
Structures of the bengazoles, bengamides and **1** isolated from *Stelletta* sp. and the proposed enzymatically controlled condensation reaction between D-isoleucine and 4-*S*-hydroxy-d-proline to yield **1**.

The DKP cyclo-(4-*S*-hydroxy-*R*-proline-*R*-isoleucine) **1** was isolated and the molecular formula C_11_H_18_N_2_O_3_, corresponding to four double-bond equivalents, was determined by (+)-ESI-FTMS accurate mass measurement. The ^13^C NMR data of **1** contained resonances consistent with the presence of two amide carbonyl groups (δ_C_ 170.5 (C-7), 165.4 (C-1)) as the only multiple bonds within the molecule, and a hydroxy methine (δ_C_ 66.8 (C-4); δ_H_ 4.28, 1H, dd, *J =* 4.6, 4.6 Hz) (Table 1). These functionalities accounted for all of the oxygen and nitrogen atoms and all of the multiple bonds in **1**, indicating the molecule to be bicyclic. Analysis of the COSY NMR data of **1** showed an extended ^1^H–^1^H spin system from H-9 to H_3_-12 via H-10 and H-11, as well as a vicinal COSY NMR correlation from H-10 to H_3_-13. Observed gHMBC NMR correlations from δ_H_ 7.97 to the ^13^C NMR resonances at δ_C_ 59.1 (C-9), δ_C_ 56.7 (C-6), δ_C_ 34.8 (C-10) and C-1 positioned this hydrogen at N-8. Further gHMBC NMR correlations from δ_H_ 4.00 (H-9) to δ_C_ 23.9 (C-11) and δ_C_ 14.9 (C-13), as well as to C-1 and C-10, clearly positioned H-9 adjacent to the C-1 carbonyl and N-8, giving rise to an isoleucine moiety (**1A**). Additional gHMBC NMR correlations from 8-NH and H-9 to C-7 revealed it was attached to N-8. A further contiguous ^1^H–^1^H spin system from H-6 to H_2_-3, in addition to gHMBC NMR correlations from the 8-NH and H_b_-3 to C-6, and from H_b_-3 to C-1 established the remaining nitrogen (N-2) to be attached to C-1, C-6 and C-3, giving rise to the two rings within **1**. The planar structure of **1** is as shown (Scheme 1).

**Figure 1:**
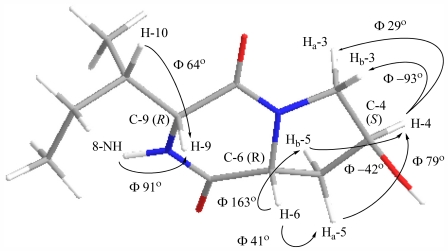
Minimum energy conformation of **1** obtained from MM2 calculations without applying any dihedral angle constraints [23]. The calculated dihedral angles for H_b_-3–C-3– C-4–H-4 (−93°), H-4–C-4–C-4–H_a_-5 (79°) and for 8-NH–N-8–C-9–H-9 (91°), all which approximate 90° as observed experimentally from the ^1^H–^1^H coupling constants, are indicative of the absolute configurations at C-4 as being *S* and at both C-6 and C-9 being *R*.

The configuration at C-4, C-6 and C-9 of **1** was established through analysis of ^1^H–^1^H coupling constants, optical rotation measurement, molecular minimisations and comparison with literature compounds [3,6,7]. The magnitude of the coupling constants associated with H-6 (dd, *J* = 11.0, 6.1 Hz) and the observed COSY NMR correlations between H-6 and H_a/b_-5 established it to have a pseudo-axial orientation, similar to that of cyclo[l-(4-hydroxyprolinyl)-l-leucine)] (H-6, dd, *J* = 11.1, 6.1 Hz) [6]. An apparent zero coupling between H-4 (*J* = 4.6, 4.6 Hz) and H_b_-3 or H_a_-5 as evident by lack of observed COSY NMR correlations, and observed couplings to H_a_-3 (*J* = 12.5, 4.6 Hz) and H_b_-5 (*J* = 12.9, 11.0, 4.6 Hz), was indicative of H-4 being orientated at approximately 90° to both H_b_-3 and H_a_-5. The observed weak COSY NMR correlation between 8-NH and H-9, and the broad singlet for H-9 (similar to that observed in cyclo-[*S*-proline-*S*-isoleucine)] [3]), revealed H-9 to be axial. Molecular modelling studies showed that the observed coupling constants were in agreement with either *R*,*R* (Figure 1) or *S*,*S* configuration at C-6/C-9 but definitely not *R*,*S* or *S*,*R* (Supplementary Data S6-S13 and Table S1). Based on optical rotation trends of DKPs from the literature [3,7], the overall positive [α]^21^_D_ = +12° indicated the absolute configuration at C-6 should be *R*, therefore supporting the *R*,*R* configuration. The magnitude of the optical rotation is also in agreement with other C-4 hydroxylated DKPs [5,7]. The molecular model shown in Figure 1, with calculated dihedral angles for H_a_-5–C-5–C-6–H-6 (Φ = 41°), H_b_-5–C-5–C-6–H-6 (Φ = 163°), H-4–C-4–C-5–H_a_-5 (Φ = 79°), H-4–C-4–C-5–H_b_-5 (Φ = −42°), H_a_-3–C-3–C-4–H-4 (Φ = 29°), H_b_-3–C-3–C-4–H-4 (Φ = −93°) and 8-NH–N-8–C-9–H-9 (Φ = 91°), best explained the observed COSY NMR correlations, ^1^H–^1^H coupling constants and the positive sign of [α]^21^_D_ confirmed the absolute configuration at C-3, C-6 and C-8 to be as shown. It is likely that this DKP was produced by an enzymatically controlled condensation reaction between d-isoleucine and 4-*S*-hydroxy-d-proline (Scheme 1) [22].

The cytotoxicity of **1**–**8** was investigated against the human tumour cell lines H460, SF-268, MCF-7, HT-29 and a normal mammalian cell line CHO-K1. The DKP **1** exhibited minimal activity towards MCF-7, H460 and HT-29 cells and no activity towards SF-268 or CHO-K1 cells at the highest dose (Table 2). In contrast, the GI_50_ values (μM) for bengamides A (**2**), F (**3**), N (**4**), Y(**5**), and bengazoles Z (**6**), C_4_ (**7**) and C_6_ (**8**) were comparable to those reported in previous studies [19,20], and accounted for the activity observed in the original MeOH extract.

## 3. Experimental

### 3.1. General Experimental Procedures

General experimental details have been described previously [29].

### 3.2. Animal Material

This specimen of the sponge *Stelletta* sp., (Family Ancorinidae) was collected from the west side of Jamieson Reef, Bonaparte Archipelago, North West Western Australia, at depths ranging from 16 m to 20 m, in August 1991. A voucher specimen (Accession number QMG312281) has been lodged with the Queensland Museum.

### 3.3. Bioassay

Cellular bioassays were undertaken as previously described [19].

### 3.4. Extraction and Isolation

Freeze dried sponge material (125 g dry weight) was extracted sequentially with dichloromethane (CH_2_Cl_2_), MeOH and H_2_O; activity was confined to the CH_2_Cl_2_ and MeOH fractions. The MeOH fraction was subjected to reversed phase C18 flash vacuum chromatography (RP-C18, 40%, 60%, 80%, 100% MeOH in H_2_O, and 100% CH_2_Cl_2_) with activity located in the 40% and 100% MeOH fractions. The 100% MeOH fraction was further separated using RP HPLC (4 mL/min, gradient elution from 60% acetonitrile (CH_3_CN):H_2_O (+0.1% formic acid [HCO_2_H]) to 100% CH_3_CN (+0.1% HCO_2_H) over 10 min, then isocratic 100% CH_3_CN (+0.1% HCO_2_H) for 15 min through a 150 mm × 10 mm 5 μ Phenomenex Luna C18 column), to give thirteen fractions. The first active fraction, fraction 1, was subjected to RP HPLC (4 mL/min, gradient elution from 20% CH_3_CN:H_2_O (+0.1% HCO_2_H) to 100% CH_3_CN (+0.1% HCO_2_H) over 20 min through a 150 × 10 mm 5 μ Phenomenex Luna Phenyl-Hexyl column) to yield bengamide Y (**5**) (0.8 mg, 0.0006%). The additional active fractions 3 and 4 were both partitioned with *n*-hexane and MeOH (1:1) to yield bengamides N (**4**) (1.4 mg, 0.001%) and A (**2**) (3.3 mg 0.003%), respectively.

The 40% MeOH fraction was subjected to further RP-C18 (10%, 20%, 30%, 40%, 50% and 100% MeOH in H_2_O) and the active fractions (30% and 40% MeOH) fractionated on RP HPLC (4 mL/min, gradient elution from 10% CH_3_CN:H_2_O (+0.1% HCO_2_H) to 64% CH_3_CN:H_2_O (+0.1% HCO_2_H) over 12 min, then isocratic 100% CH_3_CN (+0.1% formic acid) for an additional 5 min through a 150 mm × 10 mm 5 μ Phenomenex Luna C18 column) to yield bengamide F (**3**, 2.1 mg, 0.002%), bengazoles Z (**6**, 5.0 mg, 0.004%), C_4_ (**7**) (13.8 mg, 0.011%) and C_6_ (**8**) (23.6 mg, 0.012%) and the new DKP cyclo-(4-*S*-hydroxy-*R*-proline-*R*-isoleucine) **1** (1.5 mg, 0.001%).

#### 3.4.1. Cyclo-(4-*S*-hydroxy-*R*-proline-*R*-isoleucine) (**1**)

Isolated as a colourless oil. [α]^21^_D_ +12° (*c* 0.025, CHCl_3_); IR (film) *ν*_max_ 3391, 1649 cm^−1^; UV (PDA, CH_3_CN/H_2_O) λ_max_ 220 nm; ^1^H (600 MHz, *d*_6_-DMSO) and ^13^C (150 MHz, *d*_6_-DMSO) NMR data see Table 1; ESI-FTMS [M + Na]^+^ 249.1203 (calcd. for C_11_H_18_N_2_O_3_Na 249.1215).

#### 3.4.2. Bengamide A (**2**)

Isolated as a colourless oil. ^1^H NMR and ^13^C NMR spectral data were consistent with published values [17].

#### 3.4.3. Bengamide F (**3**)

Isolated as a colourless oil. ^1^H NMR and ^13^C NMR spectral data were consistent with published values [18].

#### 3.4.4. Bengamide N (**4**)

Isolated as a colourless oil. ^1^H NMR and ^13^C NMR spectral data were consistent with published values [19].

#### 3.4.5. Bengamide Y (**5**)

Isolated as a colourless oil. ^1^H NMR and ^13^C NMR spectral data were consistent with published values [20].

#### 3.4.6. Bengazole Z (**6**)

Isolated as a colourless oil. ^1^H NMR and ^13^C NMR spectral data were consistent with published values [20].

#### 3.4.7. Bengazole C_4_ (**7**)

Isolated as a colourless oil. ^1^H NMR and ^13^C NMR spectral data were consistent with published values [21].

#### 3.4.8. Bengazole C_6_ (**8**)

Isolated as a colourless oil. ^1^H NMR and ^13^C NMR spectral data were consistent with published values [21].

## 4. Conclusion

The DKP cyclo-(4-*S*-hydroxy-*R*-proline-*R*-isoleucine) (**1**), together with the known bengamides; A (**2**), F (**3**), N (**4**), Y (**5**), and bengazoles; Z (**6**), C_4_ (**7**) and C_6_ (**8**), was isolated from the Australian marine sponge *Stelletta* sp. Interestingly, this is the first report of bengamides or bengazoles from the genus *Stelletta*, however, it should be noted that they have previously been reported from species of *Dorypleres splendens* [24], which has since been reclassified as *Jaspis splendens*, and from *Jaspis* sp. [24], both of which belong to the Ancorinidae family of sponges. The cyclo-(4-*S*-hydroxy-*R*-proline-*R*-isoleucine) (**1**) was not cytotoxic against the cell lines MCF-7, H460, HT-29, SF-268 or CHO-K1. The DKP class of compounds has recently gained interest in drug discovery [25] due to their chiral, rigid and functionalised structures. These features enable them to bind to a large variety of receptors with high affinity giving rise to a broad range of biological activities, including cytotoxicity, quorum sensing, antibacterial, antifungal, antiviral, antiprion, antitumor, and immunosuppressive functions, even plant-growth regulators [7,26–28]. Our report adds to the vast knowledge of these potentially therapeutic molecules.

## Supplementary Material



## Figures and Tables

**Table 1:** NMR data for **1** (600 MHz, *d*_6_-DMSO), cyclo-[*S*-proline-*S*-isoleucine)] (300 MHz, CDCl_3_) and ^1^H NMR data for cyclo[l-(4-hydroxyprolinyl)-l-leucine)] (300 MHz, CD_3_OD).

No.	^13^C δ (m)	^1^H δ (m, *J* Hz)	COSY	gHMBC	^1^H δ (m, *J* Hz) of cyclo-[*S*-proline-*S*-isoleucine)] [3]	^1^H δ (m, *J* Hz) of cyclo[l-(4-hydroxyprolinyl)-l-leucine)] [6]
1	165.4 (s)					
2						
3	53.8 (t)	3.51 (1H, dd, 12.5, 4.6) 3.20 (1H, d, 12.5)	H_b_-3, H-4 H_a_-3	C-1, C-4, C-5, C-6 C-1, C-4, C-5, C-6	3.6–3.5 (2H, m)	3.65 (1H, dd, 12.5, 4.3) 3.42 (1H, d, 12.5)
4	66.8 (d)	4.28 (1H, br dd, 4.6, 4.6)	H_a_-3, 4-OH, H_b_-5	C-3, C-6	2.0–1.9 (1H, m) 1.9–1.8 (1H, m)	4.28 (1H, t, 4.3)
4-OH		5.10 (OH, br s,)	H-4		-	-
5	37.2 (t)	2.03 (1H, dd, 12.9, 6.1) 1.88 (1H, ddd, 12.9, 11.0, 4.6)	H_b_-5, H-6 H-4, H_a_-5, H-6	C-3, C-4 C-4, C-6, C-7	2.3–2.2 (1H, m) 2.1–2.0 (1H, m)	2.27 (1H, dd, 13.3, 6.5) 2.08 (1H, ddd, 13.3, 11.1, 4.3)
6	56.7 (d)	4.31 (1H, dd, 11.0, 6.1)	H_2_-5	C-5, C-7	4.07 (1H, t, 7.5)	4.51 (1H, dd, 11.1, 6.5)
7	170.5 (s)					
8-NH		7.97 (1H, s)	H-9	C-1, C-6, C-7, C-9, C-10	5.99 (1H, br s)	exchangeable
9	59.1 (d)	4.00 (1H, br s)	8-NH (w), H-10	C-1, C-7, C-10, C-11, C-13	3.96 (1H, br s)	4.15 (1H, m)
10	34.8 (d)	2.01 (1H, m)	H-9, H_b_-11, H_3_-13	C-1, C-13, C-11	2.4–2.3 (1H, m)	1.90 (1H, m) 1.50 (1H, dd, 8.0)
11	23.9 (t)	1.32 (1H, qdd, 11.8, 7.4, 4.5) 1.26 (1H, qdd, 11.8, 9.2, 7.2)	H_b_-11, H_3_-12 H-10, H_a_-11, H_3_-12	C-9, C-10, C-12, C-13 C-9, C-10, C-12, C-13	1.5–1.4 (1H, m) 1.3–1.1 (1H, m)	1.88 (1H, m)
12	12.3 (q)	0.82 (3H, t, 7.4)	H_2_-11	C-10, C-11	0.92 (3H, t, 7.4)	0.95 (3H, d, 6.4)
13	14.9 (q)	0.97 (3H, d, 7.0)	H-10	C-9, C-10, C-11	1.05 (3H, d, 7.2)	0.96 (3H, d, 6.4)

**Table 2:** GI_50_ (μM) data for compounds **1**–**8** against SF-268, MCF-7, H460, HT-29 and CHO-K1.

No.	SF-268 a	MCF-7 b	H460 c	HT-29 d	CHO-K1 e
**1**	>295	204	234	270	>295
**2**	<0.02	<0.02	<0.02	<0.02	0.1
**3**	1.8	0.7	0.6	1.5	32
**4**	<0.02	<0.02	<0.02	<0.02	0.2
**5**	72	52	25	48	>184
**6**	22	18	8	13	94
**7**	0.3	0.8	0.1	0.6	1.2
**8**	0.02	0.06	<0.02	0.1	0.8

a SF-268 Central nervous system-glioblastoma cells;

b MCF-7 Breast-pleural effusion adenocarcinoma cells;

c H460 Lung-large cell carcinoma cells;

d HT-29 Colon-recto-sigmoid colon adenocarcinoma cells;

e CHO-K1 Sub-clone of Chinese hamster ovary cells.
