# Supplementary material for: A New Diketopiperazine, Cyclo-(4-S-hydroxy-R-proline-R-isoleucine), from an Australian Specimen of the Sponge Stelletta sp
Source: Mar Drugs. 2011 Nov 22;9(11):2469–78. doi: 10.3390/md9112469 (PMC3229245; doi:10.3390/md9112469)
Supplement: Supplementary file 1 [file marinedrugs-09-02469-s001.pdf]

Supporting Information for

A New Diketopiperazine, Cyclo-(4-*S*-Hydroxy-*R*-Proline-*R*-Isoleucine), from an Australian Specimen of the Sponge *Stelletta* Sp.

Simon P. B. Ovenden,[†] Jonathan L. Nielson,[‡] Catherine H. Liptrot,[§] Richard H. Willis,[⊥] Dianne M. Tapiolas,[⊥] Anthony D. Wright[‖] and Cherie A. Motti[⊥,]*

Australian Institute of Marine Science, PMB no. 3, Townsville MC, Townsville, 4810, Australia

Current address: Defence Science & Technology Organisation, 506 Lorimer St. Fishermans Bend, Victoria, 3207, Australia

Current address: ACD Labs UK, Building A, Trinity Court, Wokingham Road, Bracknell, Berkshire, RG42 1PL, England

Current address: James Cook University, Townsville, QLD 4811, Australia.

Current address: University of Hawaii at Hilo, College of Pharmacy, 34 Rainbow Drive, Hilo, Hawaii 96720, USA.

[*] To whom correspondence should be addressed: Tel.: +61 7 4753 4143. Fax.: +61 7 4772 5852. Email: c.motti@aims.gov.au. [†]*Defence Science & Technology Organisation,* [‡]*ACD Labs UK,* [§]*James Cook University,* [⊥]*Australian Institute of Marine Science,* [‖]*University of Hawaii at Hilo.*

Table of Contents.

Page No

Figure S1. ^{1}H NMR spectrum (600 MHz) of cyclo-(4-*S*-hydroxy-*R*-proline-*R*-isoleucine) (**1**) in DMSO-d_6.. 3

Figure S2. ^{13}C NMR spectrum (600 MHz) of cyclo-(4-*S*-hydroxy-*R*-proline-*R*-isoleucine) (**1**) in DMSO-d_6... 4

Figure S3. COSY spectrum (600 MHz) of cyclo-(4-*S*-hydroxy-*R*-proline-*R*-isoleucine) (**1**) in DMSO-d_6.. 5

Figure S4. HSQC spectrum (600 MHz) of cyclo-(4-*S*-hydroxy-*R*-proline-*R*-isoleucine) (**1**) in DMSO-d_6.. 6

Figure S5. HMBC spectrum (600 MHz) of cyclo-(4-*S*-hydroxy-*R*-proline-*R*-isoleucine) (**1**) in DMSO-d_6... 7

Figure S6. Molecular model of (**1**) with SRR configuration……………………………………………………………………..…. 8

Figure S7. Molecular model of (**1**) with RRR configuration…………………………………………………………………...…... 8

Figure S8. Molecular model of (**1**) with RSS configuration……………………………………………………………………….... 9

Figure S9. Molecular model of (**1**) with SSS configuration…………………………………………………………………..………. 9

Figure S10. Molecular model of (**1**) with SSR configuration……………………………………………………………………………. 10

Figure S11. Molecular model of (**1**) with RSR configuration……………………………………………………………………………. 10

Figure S12. Molecular model of (**1**) with SRS configuration……………………………………………………………………………. 11

Figure S13. Molecular model of (**1**) with RRS configuration……………………………………………………………………………. 11

Table S1. Comparison of calculated dihedral angles from the eight possible stereoisomers of (**1**) with observed ^{1}H-^{1}H couplings from the ^{1}H NMR of (**1**)……..…. 12

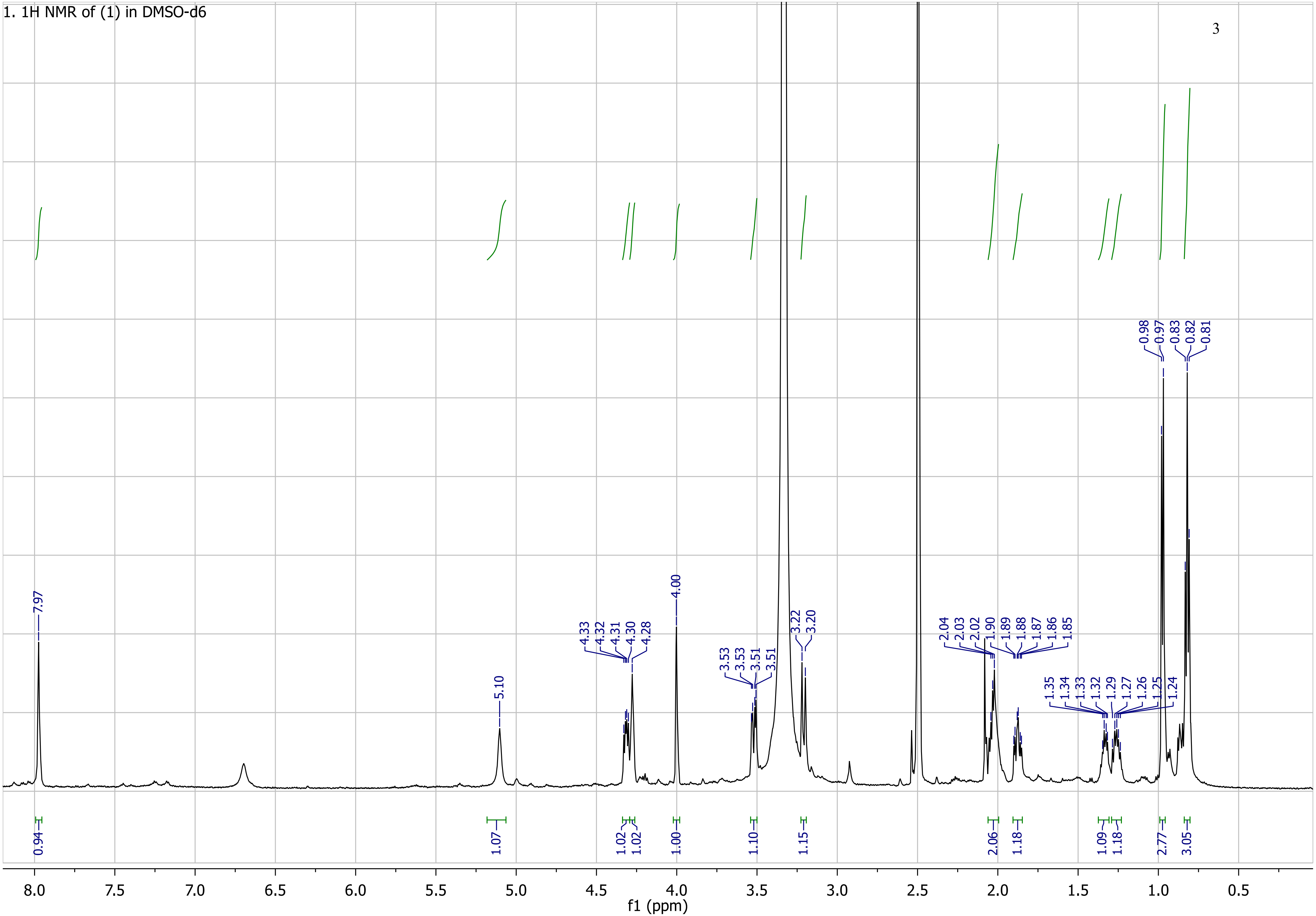
1. 1H NMR of (1) in DMSO-d6
7.97
5.10
4.33 4.32 4.31 4.30 4.28
4.00
3.53 3.53 3.51 3.51
3.22 3.20
2.04 2.03 2.02 1.90 1.89 1.88 1.87 1.86 1.85
1.35 1.34 1.33 1.32 1.29 1.27 1.26 1.25 1.24
0.98 0.97 0.83 0.82 0.81
0.94 1.07 1.02 1.02 1.00 1.10 1.15 2.06 1.18 1.09 1.18 2.77 3.05
8.0 7.5 7.0 6.5 6.0 5.5 5.0 4.5 4.0 3.5 3.0 2.5 2.0 1.5 1.0 0.5
f1 (ppm)

2. 13C NMR of (1) in DMSO-D6

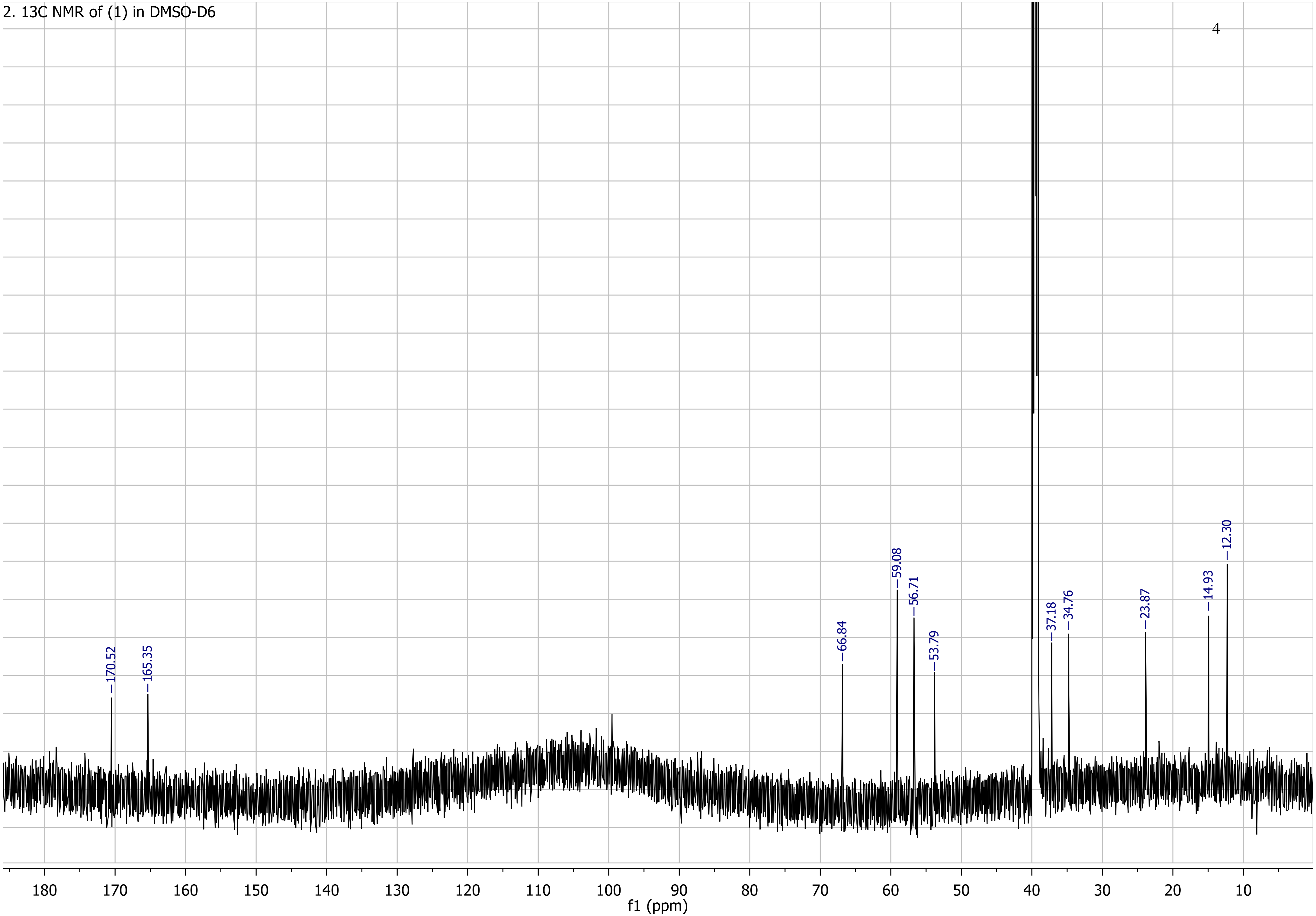

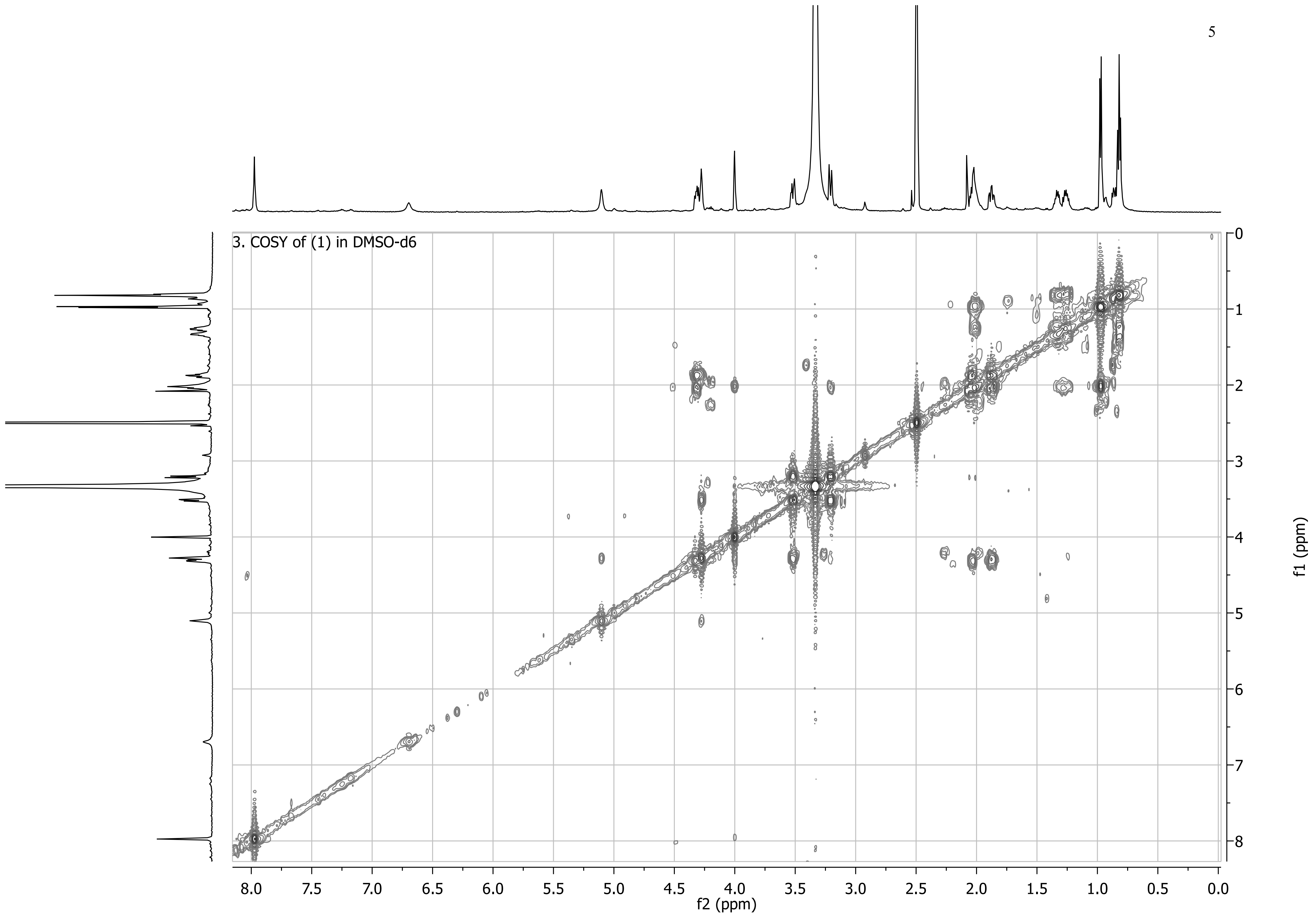
3. COSY of (1) in DMSO-d6
f2 (ppm)
f1 (ppm)
8.0
7.5
7.0
6.5
6.0
5.5
5.0
4.5
4.0
3.5
3.0
2.5
2.0
1.5
1.0
0.5
0.0
0
1
2
3
4
5
6
7
8

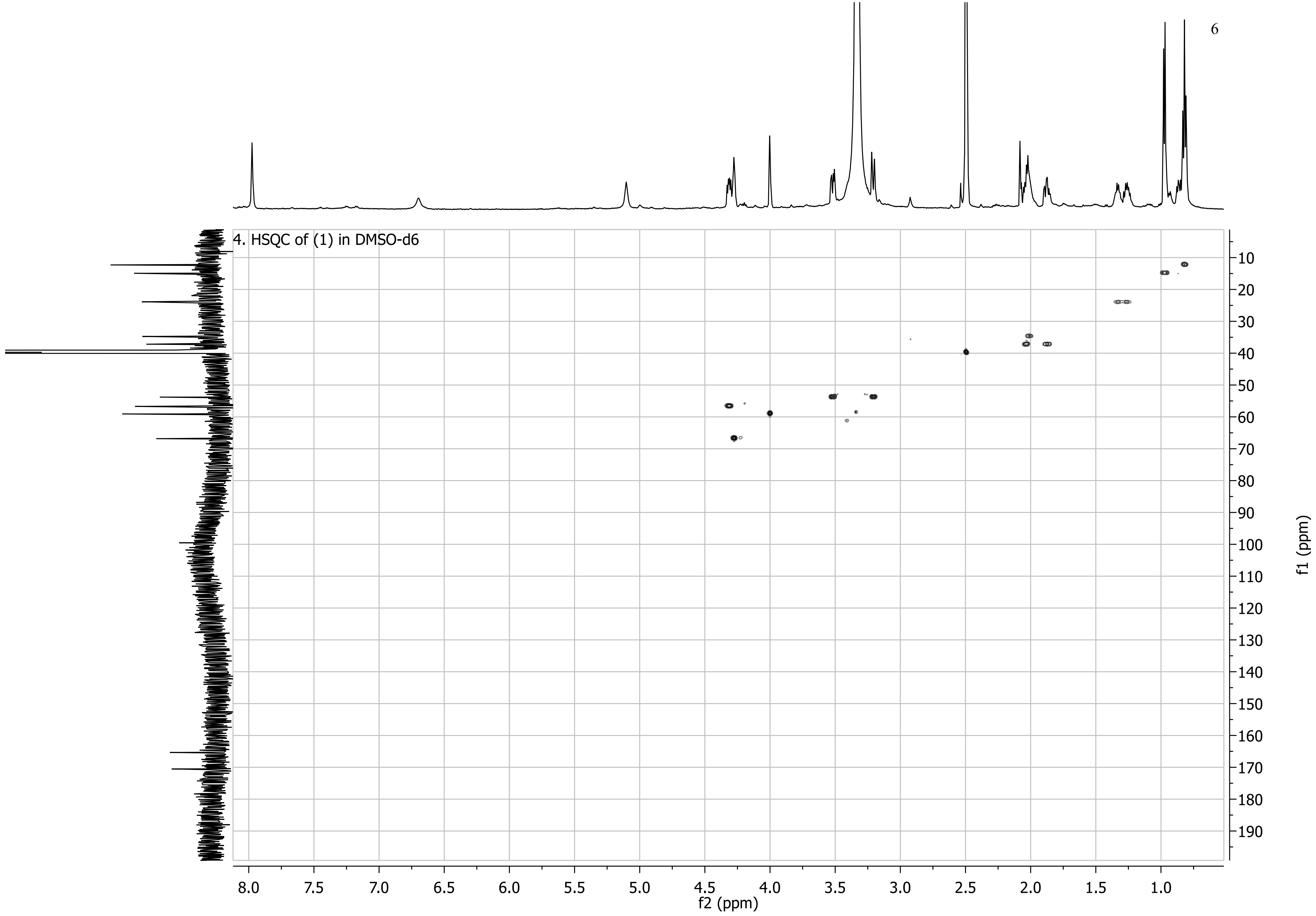
4. HSQC of (1) in DMSO-d6
f2 (ppm)
f1 (ppm)
8.0
7.5
7.0
6.5
6.0
5.5
5.0
4.5
4.0
3.5
3.0
2.5
2.0
1.5
1.0
10
20
30
40
50
60
70
80
90
100
110
120
130
140
150
160
170
180
190

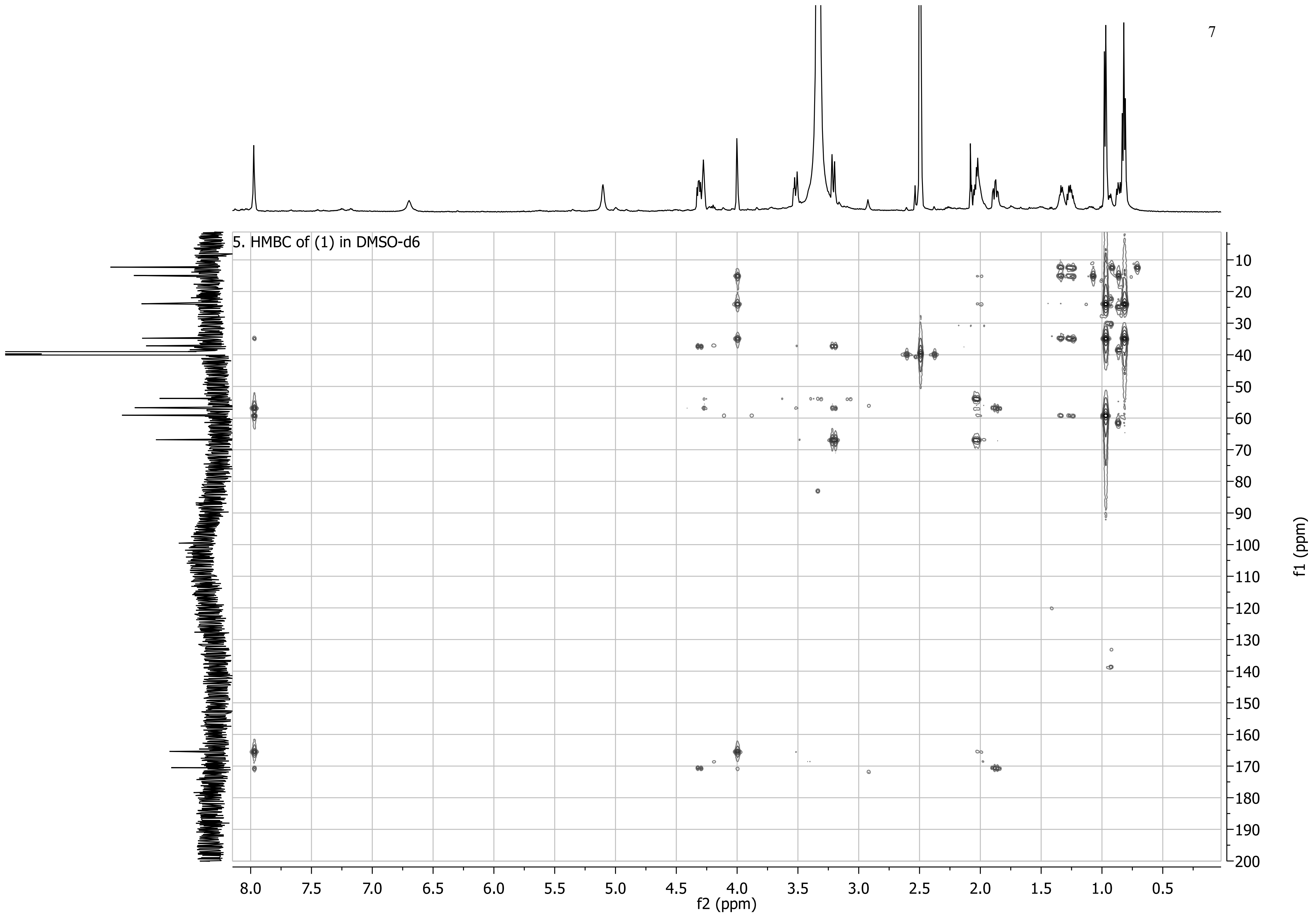
5. HMBC of (1) in DMSO-d6
f2 (ppm)
f1 (ppm)

Figure S6. Molecular model of (**1**) with SRR configuration

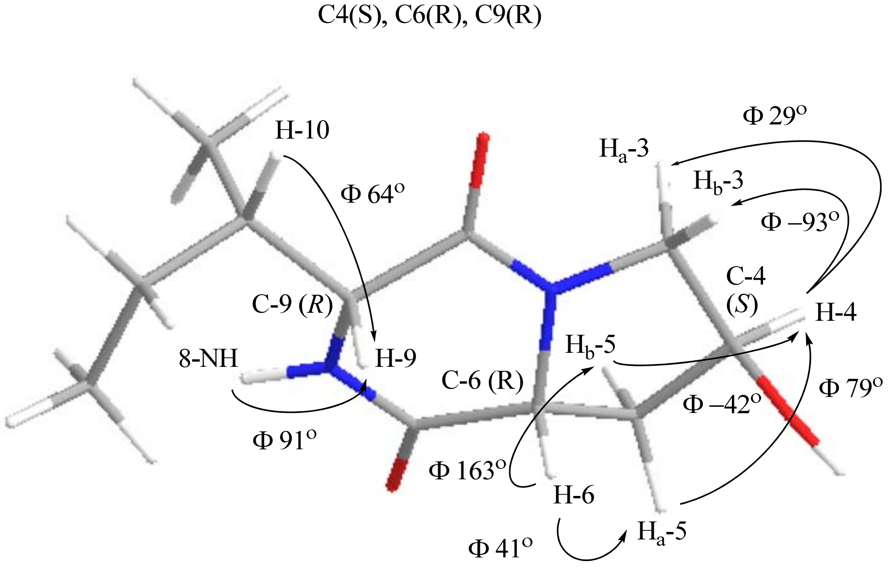

Figure S7. Molecular model of (**1**) with RRR configuration

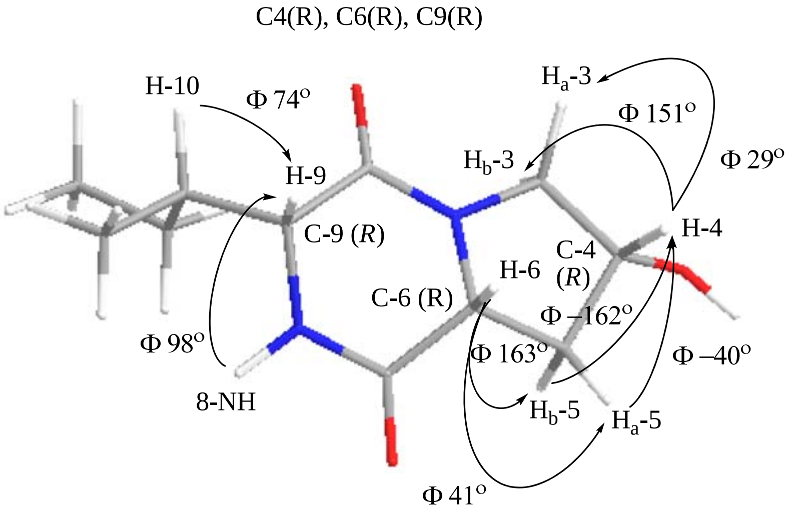

Figure S8. Molecular model of (**1**) with RSS configuration

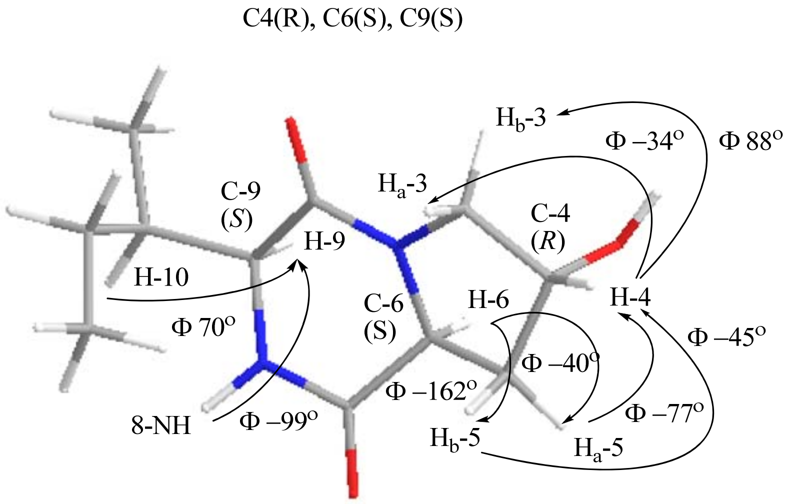

Figure S9. Molecular model of (**1**) with SSS configuration

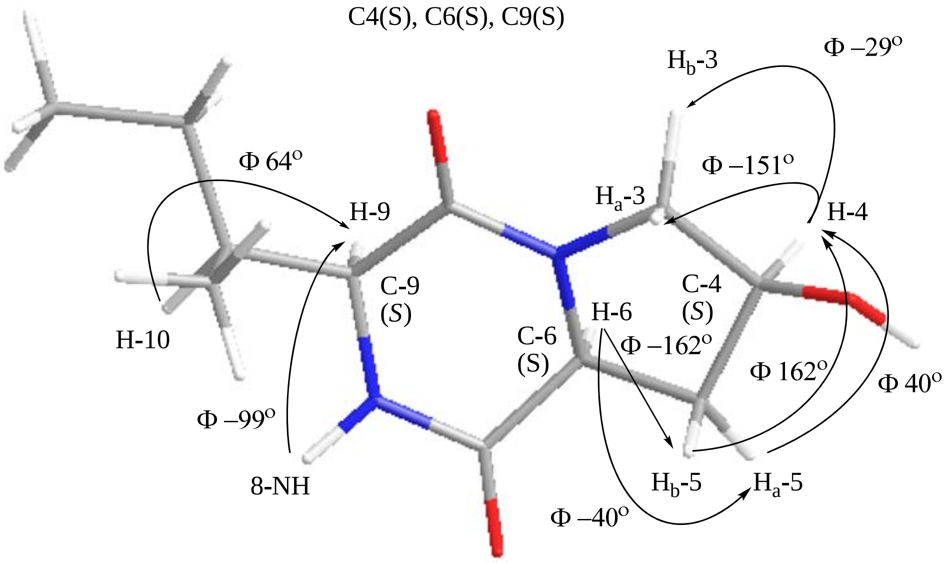

Figure S10. Molecular model of (**1**) with SSR configuration

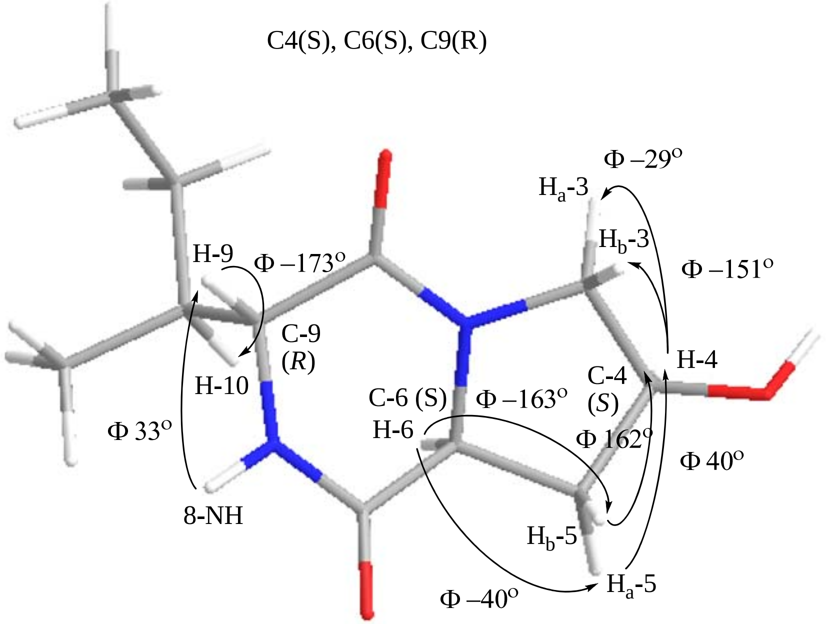

Figure S11. Molecular model of (**1**) with RSR configuration

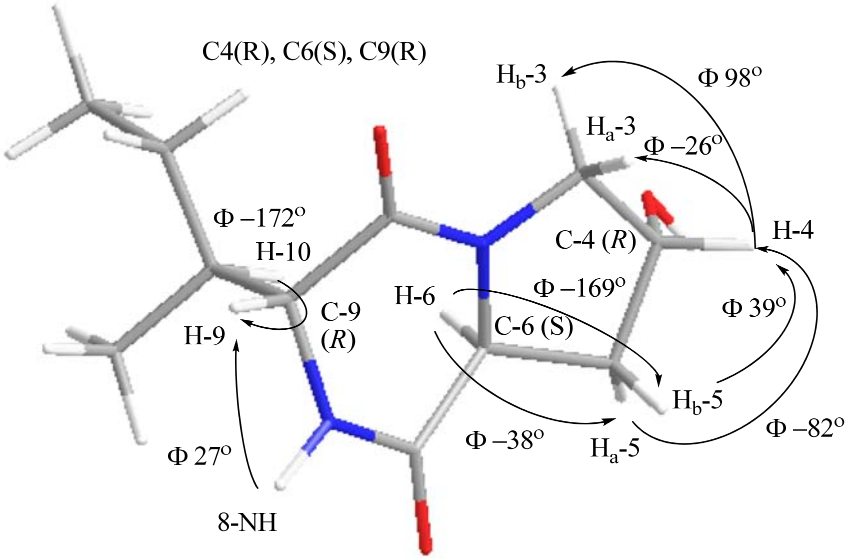

Figure S12. Molecular model of (**1**) with SRS configuration

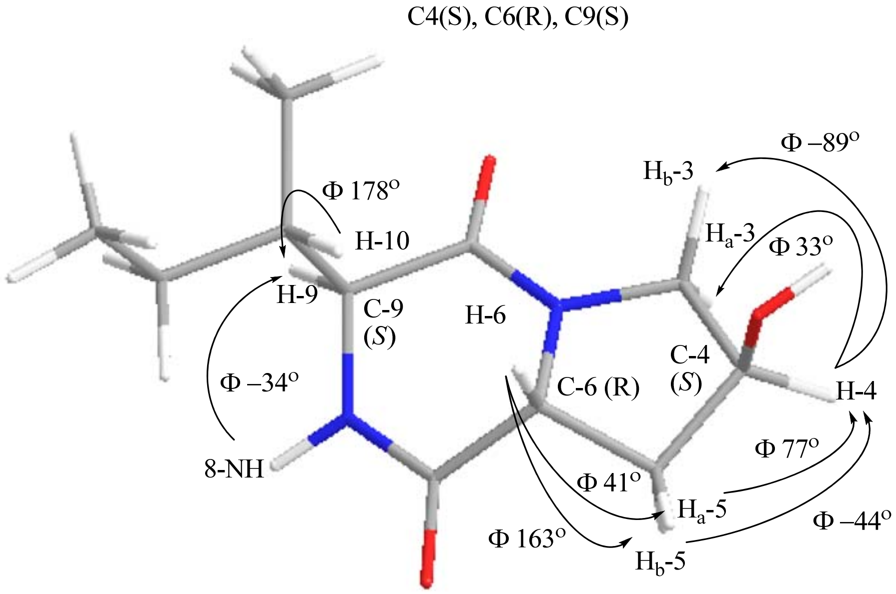

Figure S13. Molecular model of (**1**) with RRS configuration

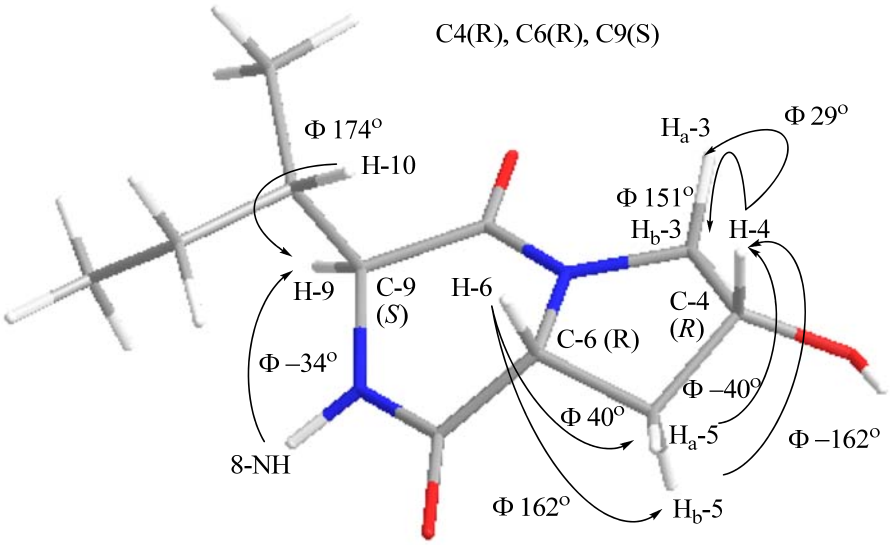

Table S1. Comparison of calculated dihedral angles from the eight possible stereoisomers of **1** with observed ^{1}H-^{1}H couplings from the ^{1}H NMR of **1**.

C-4,C-6,C-9 configuration	^{1}H-^{1}H Correlation	Calculated dihedral angle Φ	Calculated *J* (Hz) [a]	Relevant observed ^{1}H-^{1}H couplings	C-4,C-6,C-9 configuration	^{1}H-^{1}H Correlation	Calculated dihedral angle Φ	Calculated *J* (Hz) [a]	Relevant observed ^{1}H-^{1}H couplings
RRR	H-9 – 8NH	98	0.74 [b]	8NH (br s), H-9 (br s)	RSR	H-9 – 8NH	27	6.88 [b]	NA
	H-9 – H-10	74	1.16	H-9 (br s)		H-9 – H-10	-172	10.16	NA
	H-6 – H5b	163	11.26	H-6 – H5b (11.0 Hz)		H-6 – H5b	-169	11.65	H-6 – H5b (11.0 Hz)
	H-6 – H5a	41	5.63	H-6 – H5a (6.1 Hz)		H-6 – H5a	-38	6.09	H-6 – H5a (6.1 Hz)
	H-4 – H5a	-162	10.00	NA		H-4 – H5a	-82	1.50	No observed coupling
	H-4 – H5b	-40	7.14	NA		H-4 – H5b	39	4.83	H-4 (4.6, 4.6 Hz), H-5b (4.6 Hz)
	H-4 – H3a	151	8.37	NA		H-4 – H3a	-26	6.09	H-4 (4.6, 4.6 Hz), H-3a (4.6 Hz)
	H-4 – H3b	29	8.54	NA		H-4 – H3b	98	1.57	No observed coupling
SRR	H-9 – 8NH	91	0.42 [b]	8NH (br s), H-9 (br s)	SSR	H-9 – 8NH	33	6.09 [b]	NA
	H-9 – H-10	64	1.93	H-9 (br s)		H-9 – H-10	-173	10.22	NA
	H-6 – H5b	163	11.26	H-6 – H5b (11.0 Hz)		H-6 – H5b	-163	11.26	H-6 – H5b (11.0 Hz)
	H-6 – H5a	41	5.63	H-6 – H5a (6.1 Hz)		H-6 – H5a	-40	5.78	H-6 – H5a (6.1 Hz)
	H-4 – H5a	79	1.70	No observed coupling		H-4 – H5a	40	7.14	NA
	H-4 – H5b	-42	4.36	H-4 (4.6, 4.6 Hz), H-5b (4.6 Hz)		H-4 – H5b	162	10.00	NA
	H-4 – H3a	29	5.65	H-4 (4.6, 4.6 Hz), H-3a (4.6 Hz)		H-4 – H3a	-29	8.54	NA
	H-4 – H3b	-93	1.43	No observed coupling		H-4 – H3b	-152	8.51	NA
RSS	H-9 – 8NH	-99	0.80 [b]	8NH (br s), H-9 (br s)	SRS	H-9 – 8NH	-34	5.95 [b]	NA
	H-9 – H-10	70	1.86	H-9 (br s)		H-9 – H-10	178	10.40	NA
	H-6 – H5b	-162	11.18	H-6 – H5b (11.0 Hz)		H-6 – H5b	163	11.26	H-6 – H5b (11.0 Hz)
	H-6 – H5a	-40	5.78	H-6 – H5a (6.1 Hz)		H-6 – H5a	41	5.63	H-6 – H5a (6.1 Hz)
	H-4 – H5a	-77	1.86	No observed coupling		H-4 – H5a	77	1.86	No observed coupling
	H-4 – H5b	-45	3.90	H-4 (4.6, 4.6 Hz), H-5b (4.6 Hz)		H-4 – H5b	-44	4.05	H-4 (4.6, 4.6 Hz), H-5b (4.6 Hz)
	H-4 – H3a	-34	4.90	H-4 (4.6, 4.6 Hz), H-3a (4.6 Hz)		H-4 – H3a	33	5.05	H-4 (4.6, 4.6 Hz), H-3a (4.6 Hz)
	H-4 – H3b	88	1.43	No observed coupling		H-4 – H3b	-89	1.42	No observed coupling
SSS	H-9 – 8NH	-99	0.80 [b]	8NH (br s), H-9 (br s)	RRS	H-9 – 8NH	-34	5.95 [b]	NA
	H-9 – H-10	64	2.47	H-9 (br s)		H-9 – H-10	174	10.26	NA
	H-6 – H5b	-162	11.18	H-6 – H5b (11.0 Hz)		H-6 – H5b	162	11.18	H-6 – H5b (11.0 Hz)
	H-6 – H5a	-40	5.78	H-6 – H5a (6.1 Hz)		H-6 – H5a	40	5.78	H-6 – H5a (6.1 Hz)
	H-4 – H5a	40	4.94	NA		H-4 – H5a	-40	7.14	NA
	H-4 – H5b	162	11.31	NA		H-4 – H5b	-162	10.00	NA
	H-4 – H3a	-151	8.37	NA		H-4 – H3a	29	8.54	NA
	H-4 – H3b	-29	8.54	NA		H-4 – H3b	151	8.37	NA

[a] Predicted couplings calculated using the Karplus equation for ^{3}J H,H according to C.A.G. Haasnoot, F.A.A.M. DeLeeuw and C. Altona; *Tetrahedron* 36 (1980) 2783-2792.

[b] Predicted couplings calculated using the Karplus equation for ^{3}J HNCH according to V.F. Bystrov *Prog. NMR Spectrosc.* 10 (1976) 41-81.
